# The Effect of Unitizing Word Pairs on Recollection Versus Familiarity-Based Retrieval— Further Evidence From ERPs

**DOI:** 10.5709/acp-0196-2

**Published:** 2016-12-31

**Authors:** Siri-Maria Kamp, Regine Bader, Axel Mecklinger

**Affiliations:** Department of Psychology, Saarland University, Saarbrücken, Germany

**Keywords:** event-related potentials, associative recognition, unitization, familiarity, recollection

## Abstract

We investigated the contribution of familiarity and recollection to associative retrieval of word pairs depending on the extent to which the pairs have been unitized through task instructions in the encoding phase. Participants in the unitization condition encoded word pairs in the context of a definition that tied them together such that they were treated as a coherent new item, while in the control condition word pairs were inserted into a sentence frame in which each word remained an individual unit. Contrasting event-related potentials (ERERPs) elicited in a subsequent recognition test by old (intact) and recombined (a new combination of two words from different study pairs) word pairs, an early frontal effect, the putative ER P correlate of familiarity-based retrieval, was apparent in the unitization condition. The left parietal old/new effect, reflecting recollection-based retrieval, was elicited only in the control condition. This suggests that in the unitization condition only, familiarity was sufficiently diagnostic to distinguish old from recombined pairs, while in the control condition, recollection contributed to associative recognition. Our findings add to a body of literature suggesting that unitization of associations increases the relative contribution of familiarity to subsequent associative retrieval.

## Introduction

Within the field of cognitive psychology, the extent to and the circumstances under which two types of processes contribute to the retrieval of associative memories has recently been hotly debated: an effortful process that entails retrieval of contextual details of the study episode, named *recollection*, and a relatively automatic process without retrieval of context, named *familiarity*. Here, we addressed this question by reporting event-related potentials (ERPs) from a memory experiment in which the components of an association could either be linked together through task instructions, such that they were treated as a coherent new item, or were associatively encoded without becoming a new unit. The Introduction will first review the dual process account of recognition memory, which posits that access to an episodic representation can occur through recollection or familiarity. Then, we will review prior evidence for the idea that *unitization*—the process of integrating the components of an association into a unified whole-increases the relative contribution of familiarity to associative recognition.

### The Dual Process Account and Retrieval of Associative Memories

 The dual process account of recognition memory proposes that successful retrieval of previously learned information can occur through a context-free, relatively automatic process termed familiarity, or a context-dependent, more effortful process known as recollection (for a review, see Yonelinas, 2002). For example, the face of a person you see in a large crowd may seem familiar, but you do not remember the context in which you met this person. Alternatively, you may recollect that the name of this person is John Smith and that you met him in last week’s yoga class. The contribution of each process to recognition can be inferred from behavioral or neuronal data obtained in memory experiments. In the traditional view, recognition of single items can occur through both types of retrieval, while recognition of associations between multiple items or between an item and contextual information requires recollection. More recently, it has been suggested that familiarity can contribute to recognition memory for associations when the components of the association are integrated into a coherent, holistic representation (Yonelinas, Aly, Wang, & Koen, 2010 ; Yonelinas, Kroll, Dobbins, & Soltani, 1999). The process of integrating information into such a unified representation has been coined unitization (e.g., Graf & Schacter, 1989). 

 Unitization can occur in different ways, and recently the idea has been expressed that there is a continuum along which two items can be unitized ( Yonelinas et al., 2010). Sometimes, the components of an association are inherently or pre-experimentally unitized, such as the words comprised in a pre-existing compound word (e.g., Ahmad & Hockley, 2014) or the features of a face (Yonelinas et al., 1999). Alternatively, unitization of multiple aspects of an experience can occur through task instructions that lead to their integration into a unified whole. For example, participants may be instructed to unitize background color with object information by means of mental imagery (e.g., Diana, Yonelinas, & Ranganath, 2008), to engage interactive imagery to encode two objects described by a word pair (e.g., Rhodes & Donaldson, 2008), or to generate a new concept from arbitrary word pairs by applying a definition describing the pair as a novel compound (e.g., Quamme, Yonelinas, & Norman, 2007). Results from studies applying these different operational definitions of unitization generally converge on the idea that the relative contribution of familiarity to associative recognition increases when the constituents of the association are unitized. However, the precise patterns are not consistent between studies, as will be reviewed next. 

### The Effect of Unitization on Associative Memory Retrieval

#### Evidence from Behavioral Data

 One method of estimating familiarity and recollection is to generate parameter estimates from receiver operating characteristics (ROC) curves. This procedure entails the generation of a curve from hit rates and false alarm rates at multiple confidence levels. The shape of this curve gives an indication for the contribution of recollection and familiarity to the recognition decision and the two processes can thus be estimated accordingly (for a more detailed explanation, see Yonelinas, 1994). Yonelinas et al. (1999) applied this method to recognition memory for upright versus inverted faces. Only in the former case, in which the facial features are inherently unitized, familiarity contributed to recognition, while recollection did so in both conditions. Conversely, using ROC analyses, Ahmad and Hockley (2014) found no evidence for differential contributions of familiarity and recollection to associative recognition for pre-existing compound words versus arbitrary word pairs in a standard recognition memory task. Nevertheless, in a two-alternative forced choice task, a test format that fosters familiarity-based retrieval (e.g., Bastin & Van der Linden, 2003), there was a discrimination advantage for the compounds, supporting the idea that familiarity contributes to recognition of unitized stimuli (Ahmad & Hockley, 2014). Also using the ROC procedure, Diana et al. (2008) found that, when background color was unitized with object information by mental imagery, the contribution of familiarity to source retrieval was increased. However, the effect on recollection was equivocal: In versions of the paradigm that differed in the nature of the control task and the background color to be unitized with the object, the values of the recollection parameter increased, decreased, or were unaffected. Finally, Parks and Yonelinas (2015) compared two conditions in which word pairs (e.g., cloud-lawn) were encoded either together with a definition that tied them into a new concept (high-unitization; “A yard used for sky gazing”) or with a sentence frame that kept each word as a separate unit (low-unitization; “He watched the ___ float by as he sat on the ___.”). In the high-unitization condition, contributions of both recollection and familiarity to associative recognition were increased, resulting in a performance advantage (but see Bader, Mecklinger, Hoppstädter, & Meyer, 2010 ; Quamme et al., 2007 , for examples where performance did not differ between the same two conditions). In the same task, an advantage of the unitized pairs under familiarity-only recognition instructions (Quamme et al., 2007) and in a priming task (Parks & Yonelinas, 2015) further supports the idea that familiarity contributes more to the high- than the low-unitization condition. Note, however, that these studies did not allow for conclusions about the effect of unitization on recollection. 

#### Evidence from event-related potentials

 ERPs elicited during a recognition task provide another way of measuring familiarity and recollection. A frontally distributed difference between old and new items about 300 to 500 ms after stimulus onset has been associated with familiarity-based retrieval, while a later onsetting parietal effect co-occurs with recollection (for a review, see Rugg & Curran, 2007 ; but see Paller, Voss, & Boehm, 2007 , for a different view). This distinction is supported by findings showing that the midfrontal effect increases with familiarity strength (e.g., Woodruff, Hayama, & Rugg, 2006 ; Yu & Rugg, 2010), and does not distinguish between false alarms and correct rejections to lures, while the parietal effect does (e.g., Curran, 2000). Furthermore, a response deadline procedure, which fosters familiarity-based responding, affects the parietal but not the mid-frontal old/new effect (e.g., Mecklinger, Brunnemann, & Kipp, 2011). Finally, the parietal old/new effect varies with the amount of recollected information (e.g., Vilberg, Moosavi, & Rugg, 2006). In tests of associative recognition, only the parietal effect (and sometimes additional effects following it) tends to occur (e.g., Donaldson & Rugg, 1998). The patterns have been mixed when a unitization manipulation was employed, but they generally converge on the idea that the relative contribution of familiarity to associative retrieval increases with unitization. 

 In one study (Jäger, Mecklinger, & Kipp, 2006), face pairs were studied that either both depicted the same, or two different, individuals. Only the former condition, in which the face parts could be unitized, elicited an early old/new effect, while the late effect was found only in the latter (non-unitization) condition. A similar double dissociation between the putative ERP correlates of familiarity and recollection has been found between the sentence and definition tasks described in the previous paragraph (Bader et al., 2010). This suggests that unitization increases familiarity-based recognition and-under some circumstances-enables this familiarity mechanism to be sufficiently diagnostic of previous presentation, so that recollection-based remembering can be bypassed. 

 By contrast, Rhodes and Donaldson (2007, 2008) found that unitization of word pairs led to an enhanced early old/new effect, but did not affect the late old/new effect. Note, however, that an enhanced late effect was found for the unitization condition when intact pairs were contrasted with recombined rather than new pairs (Rhodes & Donaldson, 2008). Similarly, using a task in which either internal or external context features of faces were task relevant, only the former representing a case of pre-experimental unitization, Guillaume and Etienne (2015) reported that the early old/new effect was increased by unitization, but the late old/new effect was unaffected. 

 Finally, using pre-existing compound words (Zheng et al., 2015) or images that were in a semantically meaningful (i.e., unitizable) pairing (Tibon, Gronau, Scheuplein, Mecklinger, & Levy, 2014), both the early and the late old/new effect have been found to be larger after unitization encoding, compared to control conditions. 

 Yet another pattern was reported in a paradigm in which word pairs were either categorically (e.g., *dancer-singer*) or thematically (e.g., *dancer-stage*) related. Although only the latter type of word pair should encourage unitization, the early frontal old/new effect was of equivalent magnitude in both conditions. However, the late old/new effect was only evident in the condition with categorically related word pairs. This indicates that familiarity may have been sufficiently diagnostic for the easy-to-integrate thematic pairs, whereas for categorically related pairs with their large feature overlap recollection is required for successful associative recognition (Kriukova, Bridger, & Mecklinger, 2013). 

Taken together, different operationalizations of unitization have in previous studies led to heterogeneous effects of unitization on behavioral and ERP measures of recollection and familiarity. Differences between these studies in experimental design and analysis, such as whether encoding was intentional or incidental, likely contributed to this heterogeneity. Nevertheless, there is some prior evidence that the relative contribution of familiarity (vs. recollection) to associative recognition is enhanced by unitization.

### The Present Study

The heterogeneity of prior findings suggests, first, that replications of the previously reported effects are desirable. Furthermore, for technical or other reasons, most prior ERP studies have contrasted old (intact) associative probes with probes in which both items of the pair were new. This contrast does not control for familiarity for each individual item of a pair, which could provide the basis for distinguishing between the two pair types but which would not require retrieval of whether the two items occurred together. In this respect, on the one hand, a better controlled contrast would be between old (intact) probes and recombinations of items previously studied as parts of different study trials because familiarity of the individual items should be equal for both pair types, so only this distinction requires the retrieval of associative information. On the other hand, it should be noted that the old versus recombined contrast has the disadvantage that recombined trials may elicit recollection of the correct pairing of one or both of the presented items as well. It is therefore ideal to consider both contrasts for a given paradigm to gain a complete understanding of the processes involved.

 To this end, we analyzed ERPs elicited during recognition of word pairs in a slightly modified paradigm of Bader et al. (2010; see also Bader, Opitz, Reith, & Mecklinger, 2014 ; Quamme et al., 2007). In the definition condition, the word pairs were defined as novel compound words that enable unitization encoding. In the sentence condition, the word pairs were relationally encoded by means of a sentence frame that kept each word as a separate entity. In each trial of the encoding phase, participants rated how much sense the new concept or sentence made to them. To center the analysis on those trials where unitization was most likely successful, we focused on only those word pairs for which participants indicated that the definition or sentence provided a good fit. 

 In extension to Bader et al. (2010), we here compare ERPs elicited by old (intact) to recombined rather than completely new pairs. Throughout the remainder of the manuscript we refer to these differences between old and recombined pairs as “intact/recombined effects”, but to the extent that they are morphologically analogous, we assume that these effects reflect similar processes as old/new effects that have been well-characterized in previous research. Our goal was to test whether this contrast would support that an enhanced early but a reduced late effect is elicited in the high unitization encoding (definition) condition relative to the control (sentence) condition. A between-subjects design was used to prevent strategic carry-over effects during encoding, to permit the use of an incidental encoding task, and to insure comparability to prior studies using this paradigm (Bader et al., 2010 , 2014; Parks & Yonelinas, 2015 ; Quamme et al., 2007). 

## Method

### Participants

Fourty-two young adults (ages 19-30 years), who were all native German speakers and reported to have no history of neurological conditions, took part in the study and were randomly assigned to one of two encoding conditions. There was no age difference (*p* > .64) between participants in the definition (*M*_age_ = 23.14, *SD* = 2.37, *n* = 21, 12 female, nine male) and the sentence (*M*_age_ = 23.52, *SD* = 2.91, *n* = 21, 14 female, seven male) condition.

### Stimuli and Procedure

 The stimuli were taken from Bader et al. (2014)^1^, and the procedure was modeled after this and previous studies (Quamme et al., 2007). In the encoding phase, participants incidentally encoded a sequence of 160 pairs of unrelated nouns that formed grammatically legal but not pre-existing compound words. Each trial began with the presentation, below the center of the screen, of a definition that described the associated word pair as a novel concept (definition condition), or a sentence with two blanks in which the two words of the pair could be inserted (sentence condition). After the additional presentation of a fixation cross in the center of the screen for 1 s, a word pair with five blank spaces between the two words was shown in the center of the screen for 2 s. After a 500 ms blank screen, this was followed by a rating screen prompting the participants to judge how plausible they considered the new concept or sentence, or, in other words, how well they could imagine it, on a scale of 1 (*very well*) to 4 (*very poorly*). The participant’s response terminated this screen, and a 2 s long intertrial interval separated two successive trials. 

After a 5 min long distractor task, in which participants counted backwards in steps of three, the recognition test began. Participants were presented with a random sequence of 80 intact (old) pairs from the encoding phase, as well as 80 pairs with a recombination of two words from different study pairs. Which pairs were presented as intact and recombined, respectively, was determined at random and was different for each participant. The task was to distinguish between these types of pairs and deliver the judgment on a scale of 1 (*definitely old*) to 6 (*definitely new*; note that new in the task referred to the pairings, not the individual words of a pair). In both the encoding and recognition phases, breaks were allowed after each set of 40 trials.

### EEG Recording and Analysis

 The present manuscript reports the EEG data recorded during the recognition phase of the experiment from 28 Ag/AgCl scalp electrodes with a BrainAmp (Brain Products, Inc.) DC amplifier with a 50 Hz notch filter was used. The EEG was digitized at 500 Hz. Four electrodes around the eyes recorded electro-ocular activity and electrode FCz was used as the ground. Online, the EEG was referenced to the left mastoid, and offline, it was re-referenced to linked mastoids. Using BrainVision Analyzer software, we band-pass filtered the EEG at 0.1-30 Hz. Segments including a time window of 200 ms before the onset to 800 ms after the onset of each word pair were extracted from the recognition phase, which were corrected for eye blink and saccade artifacts using the regression method implemented in BrainVision Analyzer (Gratton, Coles, & Donchin, 1983). Segments were excluded from further analysis if they contained voltage steps of 20 μV or if the difference between the maximum and the minimum value in the segment exceeded 100 μV. 

Next, we calculated individual ERP averages for old and recombined pairs, including only trials with correct recognition judgments of high or medium confidence. By using each participant’s individual rating from the encoding phase, old (intact) pairs were further divided into those where the new concept was formed by the two words together with the definition or the new sentence that resulted from mentally inserting the words into the sentence frame was judged as easy to imagine (*very well* or *rather well* judgments; referred to as “high-fit old” pairs), and those that were judged as hard to imagine (*rather poorly* or *very poorly* judgments; referred to as “low-fit old” pairs). In the present manuscript, we only report ERP results for old (intact) pairs, which were judged as high-fit old during encoding. These pairs are of main interest to our hypotheses, because unitization in the definition condition is most likely to be successful when the two words of a pair are easily integrated into a new concept. Furthermore, for several participants (*n* = 9 in the definition and *n* = 10 in the sentence condition) the low-fit old category provided less than 15 artifact-free trials. For the recombined pairs, there was no analogous subdivision into high-fit and low-fit because *fit* was defined as the extent to which participants considered the word pair to fit into the sentence or the extent to which they considered the word pair together with the definition to form a plausible new concept. However, for recombined pairs, no sentence or definition was available. Therefore, the recombined pairs were included in ERP averages independently of the fit rating provided for the different study pairs, which the two words of a recombined pair stemmed from.

Consistent with previous ERP studies on episodic memory, participants were thus included in the ERP analysis if at least 15 trials were available for each ERP average reported here. Using this criterion, ERPs from 20 and 18 subjects in the definition and sentence condition respectively entered the analysis. The mean trial numbers in the high-fit old category were 27.2 and 27.6 for the definition and sentence conditions, respectively, and in the recombined category on average 47.15 and 43.39 trials were included in the two conditions. There were no condition differences in trial numbers for either trial type (*p* > .37 for both types).

We contrasted high-fit old pairs with recombined pairs in two time windows. The early time window included 350-500 ms after pair onset, a time window that is frequently used for the quantification of the early old/new effect, the putative correlate of familiarity-based retrieval. The late time window spanned 550-750 ms after pair onset, thus covering the old/new effect indexing recollection-based retrieval.

### Statistical Analysis

The statistical analysis of ERP amplitudes in the early and late time windows focused on four electrode clusters, covering scalp areas where, based on prior research, the early and late old/new effects are typically prominent: a left frontal (electrodes F3, FC3, and FC5), a right frontal (F4, FC4, and FC6), a left parietal (P7, P3, and CP3), and a right parietal (P8, P4, and CP4) cluster. On the averaged amplitudes for these electrode clusters, we conducted 4 × 2 × 2 (Electrode Cluster [left frontal, right frontal, left parietal, right parietal] × Pair Status [old, recombined] × Condition [definition, sentence]) mixed ANOVAs. Greenhouse-Geisser corrections were applied whenever the assumption of sphericity was violated. We only report main effects of pair status and interactions involving the factors pair status or condition. Follow-up tests included lower level ANOVAs and *t*-tests.

## Results

### Behavioral Data

**Figure 1.**
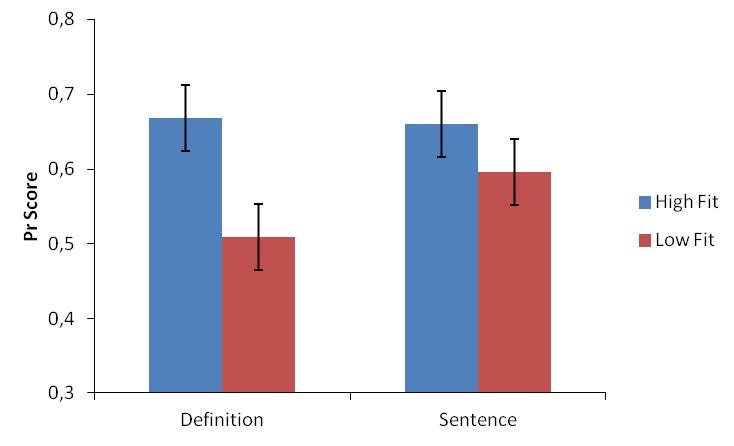
Probability of true recognition (Pr) scores, excluding responses given with low confidence, by condition and by fit rating provided during encoding. Error bars represent 95% confidence intervals for the condition by fit rating interaction (Jarmasz & Hollands, 2009).

In line with the trial types included in the ERP analysis, we calculated probability of true recognition (Pr) scores (hit rates-false alarm rates) for high- and low-fit trials separately, using only trials that were judged with high (*definitely old*) or medium confidence (*probably old*) as hits and false alarms (see Figure 1)^2^. The 2 × 2 (Condition [definition, sentence] × Fit (high, low)]) ANOVA on the Pr scores did not reveal a main effect for condition (*p* > .51), suggesting that recognition performance did not differ between the conditions. However, there was a main effect for fit, *F*(1, 40) = 25.22, *p* < .001, η_p_^2^ = .39, indicating higher recognition performance for high-fit trials, as well as a condition by fit interaction, *F*(1, 40) = 4.57, *p* = .04, η_p_^2^ = .10. Although the difference between high-and low-fit trials was significant for both conditions (*p* < .05 for both conditions), it was larger in the definition condition (see Figure 1).

### Event-Related Potentials

#### Early time window (350-500 ms)

**Figure 2.**
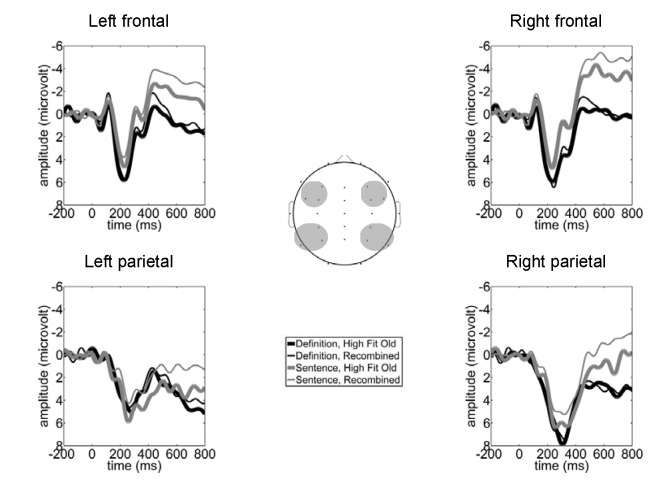
Grand average event-related potentials (ERPs) at the four electrode clusters included in the statistical analysis.

**Figure 3.**
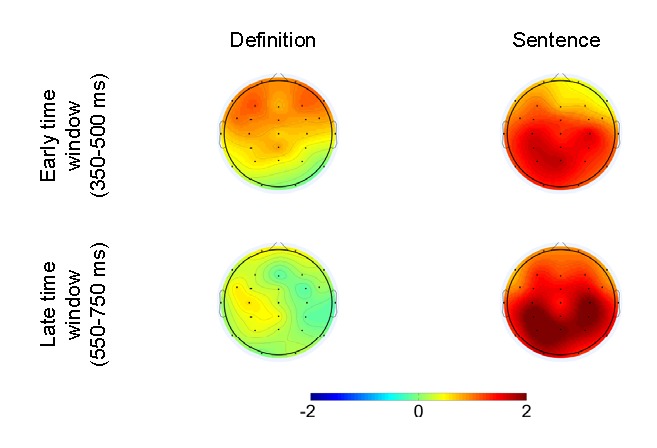
Spatial distributions of the old/recombined difference in amplitude for the early and late time windows and for both experimental conditions.

 As expected, in the early time window, high-fit old pairs elicited less negative-going amplitudes than recombined pairs (see Figure 2). This was confirmed by a significant main effect for pair status in the ANOVA, *F*(1, 36) = 8.76, *p* < .01, η_p_^2^ = .2. There was also a three-way interaction between cluster, pair status, and condition, *F*(1.81, 65.17) = 3.46, *p* = .04, η_p_^2^ = .09. For the definition condition, the early intact/recombined effect was significant (*p* < .05) at the left and right frontal electrode clusters, while for the sentence condition it was significant bilaterally at the parietal, as well as at the left frontal electrode clusters. The Electrode Cluster × Condition interaction remained significant after calculating and vector-scaling (McCarthy & Wood, 1985) the old/recombined difference, suggesting that the early intact/recombined effects in the two conditions exhibited different distributions (see Figure 3). 

#### Late time window (550-750 ms)

High-fit old pairs in the sentence but not in the definition condition elicited more positive-going amplitudes than recombined pairs (see Figures 2 and 3). This impression was substantiated by the fact that the main effect for pair status, *F*(1, 36) = 7.35, p < .01, η_p_^2^ = .17, was qualified by an interaction between pair status and condition, *F*(1, 36) = 5.06, *p* = .031, η_p_^2^ = .12. A separate 4 × 2 (Electrode Cluster [left frontal, right frontal, left parietal, right parietal] × Pair Status [old, recombined]) ANOVA, as well as additional tests at each electrode cluster alone, revealed no main effects or interactions involving the factor pair status for the definition condition (*p* > .59 for all combinations). By contrast, in the sentence condition, there was a main effect for pair status, *F*(1, 17) = 12.76, *p* < .01, η_p_^2^ = .43: ERPs elicited by high-fit old pairs were more positive-going than those elicited by recombined pairs. These results suggest that there was a late ERP difference between high-fit old and recombined pairs in the sentence but not the definition condition (see Figure 3).

#### Early versus late time window

**Figure 4.**
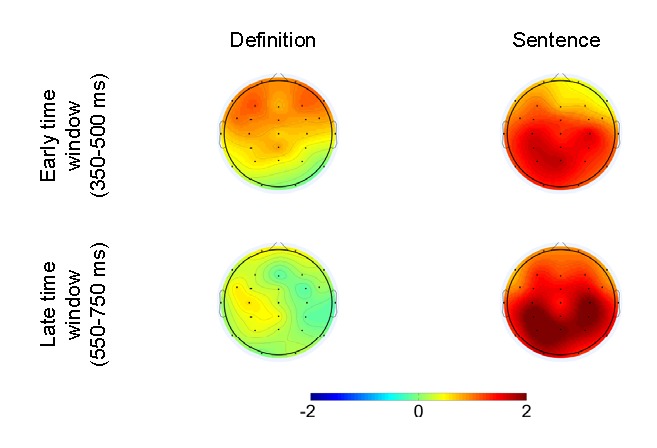
Magnitude of the early and late old/new effects at the electrode clusters where each effect was maximal.

 To summarize, in both conditions an early difference between old and recombined pairs was obtained, which exhibited qualitatively different topographic distributions for the two conditions but was significant for each condition in left frontal electrodes. By contrast, the late parietal effect was only elicited in the sentence condition. To substantiate this pattern we calculated the amplitude difference between high-fit old and recombined pairs at the left frontal electrode cluster for the early time window and at the left parietal electrode cluster for the late time window, separately for each of the encoding conditions (for a similar procedure see Bader et al., 2010). The interaction in the 2 × 2 (Effect [early, late] × Condition [definition, sentence]) ANOVA (see Figure 4) was significant, *F*(1, 36) = 4.1, *p* = .05, η_p_^2^ = .1. This interaction further supports that the conditions differed in the relative contributions of the two processes to retrieval. 

 To insure that the early effect in the definition condition and the late effect in the sentence condition indeed reflected distinct neural processes, we compared the distributions of the two effects (the early effect for the definition condition and the late effect for the sentence condition) in a 4 × 2 (Electrode Cluster [left frontal, right frontal, left parietal, right parietal] × Condition [definition, sentence]) ANOVA on the vector-normalized differences between old and recombined pairs (McCarthy & Wood, 1985). The interaction was significant, *F*(1.79, 64.59) = 3.42, *p* = .04, η_p_^2^ = .09, supporting the idea that different neural generators underlie the two effects. 

## Discussion

 Bader et al. (2010) reported that when word pairs are tied into a unified concept by means of a definition describing them as novel compound words, an early old/new effect is enhanced while at the same time the late old/new effect is reduced, compared to relational encoding through the use of a sentence in which the words of the pair could be inserted. This suggests that unitization encoding leads to a familiarity signal, which is sufficiently diagnostic for the prior occurrence of an association, so that recollection is not required for associative recognition and can be circumvented. However, Bader et al. (2010) only reported an ERP contrast between old pairs and pairs of completely new items. In the present study, we extended these previous results by contrasting old with recombined, rather than with new pairs, to determine whether the respective ERP effects were truly diagnostic of associative recognition. Indeed, in the absence of behavioral differences between the two conditions, the ERP analyses revealed an early intact/recombined effect with a frontal maximum in the definition condition, suggesting that familiarity could be used to distinguish old from recombined pairs. Additionally and somewhat unexpectedly, an effect with a parietal maximum with the time course of the early old/new effect was also pronounced in the sentence condition. Crucially, only in the sentence condition did a late parietal intact/recombined effect indicative of recollection occur. 

### The Effect of Unitizing Word Pairs on Early and Late Intact/Recombined Effects

 In combination with the findings of Bader et al. (2010) , the interaction between experimental condition and early versus late intact/recombined effect reported in the present manuscript demonstrates that the relative contributions of familiarity and recollection to associative recognition of word pairs is altered by the extent to which the study task encourages unitization of the association. By showing ERP differences between old (intact) and recombined pairings, our results also provide evidence that the familiarity and recollection signals, respectively, are truly diagnostic of the associative recognition decision: While in the old versus new contrast reported by Bader et al. (2010) it is possible that both familiarity for single items and associative information about their co-occurrence distinguished the trial types, the old versus recombined contrast in our analysis insures that item familiarity is controlled for and only associative information about the exact pairing can be used for the associative recognition judgments. 

It is worth noting that in an initial ERP analysis in which the old (intact) pairs were included regardless of the fit rating given during encoding, the results regarding the late old/recombined effect were similar: The effect was only observed in the sentence condition. By contrast, the early old/recombined effect was not significant in either the sentence or the definition condition.^3^ It therefore appears that the extent to which familiarity aids the distinction of unitized item pairs from recombined pairs depends on whether the unitization manipulation is actually successful. This idea is also supported by the effect of fit ratings on recognition performance.

 There may be some concern with the fact that our ERPs for the old (intact) pairs selectively included high-fit trials but that recombined pairs included words regardless of the associated fit rating at study. This analysis approach was chosen because for recombined pairs there is no judgment of fit that is comparable to the intact pairs: *Fit* is defined as the extent to which the pair can be integrated into a new concept, or into the new sentence, but for the recombined trials neither a new concept nor a new sentence exists. Assuming that words that stem from high-fit pairs have been processed in a qualitatively and/or quantitatively different manner, one may propose to construct high-fit recombined pairs from two words taken from separate study pairs that were each judged as highly fitting. This approach would better equate old and recombined pairs in the way each word of a pair was processed at encoding. However, Pilgrim, Murray, and Donaldson (2012) have shown that unitization may actually lead to a reduction of familiarity for the individual components of an association. Hence, focusing on such high-fit recombined trials might actually lead to an underestimation of familiarity for recombined trials as compared to our analysis, which included all trials. Furthermore, we would like to stress that the same analysis approach was followed in both conditions, so the between-condition comparison should not be biased by our analysis approach. Perhaps one way to circumvent this issue in future studies is to include in the experiment only pairs that are likely to be judged as highly fitting by participants. The resulting low number of low-fit trials may then have such a small impact that an analysis that disregards the participants’ fit ratings at encoding retains sufficient power to detect effects of unitization on measures of familiarity. 

#### Early Intact/Recombined Effect

 Unexpectedly, the two conditions did not differ in the magnitude (but in the spatial distribution) of the early intact/recombined effect. An objection could be that the present design was underpowered and a condition difference in the early effect may have been revealed with higher power. However, the presence of reliable group differences in the late intact/recombined effect, which were obtained with the same experimental design, as well as the significant interaction between the factors group and ERP effect (early vs. late) argue against this objection. Based on our data, we therefore cannot conclude that familiarity made a contribution to retrieval in the definition condition only. In this context it is important to consider again that in the ERP analysis of the present manuscript we only included old trials in which the word pairs were judged as being easily integrated into the new concept or sentence (high-fit trials). This contrast maximizes the contribution of unitization to associative retrieval because the high-fit trials should be those where the manipulation of unitization or relational encoding, respectively, worked best. However, as a result, ERPs in the sentence condition may also reflect some degree of unitization because word pairs that fit really well into a sentence (which perhaps even encourage interactive imagery) should also exhibit a relatively high likelihood of being integrated into a holistic representation. Our assumption that unitization should be stronger and more relevant for associative recognition in the definition condition is empirically supported by the fact that in the definition condition recognition performance dropped especially strongly for word pairs for which participants indicated that the definition did not provide a good fit for the new concept during encoding. However, recognition performance was also lower for low- than high-fit old trials in the sentence condition (see Figure 1), so it is likely that indeed both conditions elicit some degree of unitization. Unitization is therefore better conceived of as a continuum than an all-or-nothing process (e.g., Parks & Yonelinas, 2015 ; Yonelinas et al., 2010). 

 Although familiarity possibly contributed to recognition of word pairs in the sentence condition as well, the fact that the late intact/recombined effect was absent in the definition condition when both conditions were equivalent on recognition performance suggests that only in this condition familiarity was sufficiently diagnostic to distinguish between high-fit old and recombined pairs, while in the sentence condition recollection occurred in addition. This pattern is reminiscent of Kriukova et al. (2013) , who reported that word pairs, that, due to their integrative thematic relations, were more easily unitized (e.g., *singer-stage*), elicited an equivalent early but a reduced late old/new effect, compared to categorically related word pairs (e.g., *dancer-stage*) for which the formation of a unitized representation is difficult. Taken together, these result patterns suggest that recollection and familiarity may be independent of each other or that at least an experimental manipulation can affect recollection without affecting familiarity (see Tibon & Henson, 2015 , for a discussion; although some caution is in order when drawing such a conclusion due to the different spatial distributions of the early intact/recombined effect in the two conditions of our study, as discussed below). 

*Spatial Distribution in the Definition Condition.* In our data, the early intact/recombined effect in the definition condition exhibited a frontal maximum, while in Bader et al. (2010) the distribution of the old/new effect was parietal. To interpret this difference in scalp distribution it is useful to consult the concept of relative versus absolute familiarity (Mandler, 1980) and their reflection in early ERP effects. Bridger, Bader, and Mecklinger (2014) have reported that ERPs in an absolute familiarity contrast (i.e., high- vs. low-frequency words) differ in an N400-like component with a parietal distribution presumably reflecting enhanced baseline (absolute) familiarity of high-frequency words, their facilitated semantic processing, or a combination thereof. Conversely, ERPs elicited by repeated low-frequency words elicited a frontally distributed early old/new effect, presumably reflecting high amounts of incremental (relative) familiarity for the low-frequency words. Accordingly, the parietally distributed early old/new effect in Bader et al. (2010) has been interpreted as a reflection of enhanced absolute familiarity in the sense that for the novel concepts, which exhibit very low pre-experimental or absolute familiarity, increments in absolute familiarity are mnemonically highly diagnostic. 

 The frontal distribution of the early ERP effect reported in the present manuscript may thus be due to the old versus recombined contrast. To distinguish old from new pairs, familiarity of the single components to be unitized as well as absolute familiarity and/or fluency of the concept can be diagnostic. As already noted, due to the contribution of absolute familiarity the distribution of the ERP difference may be more parietal than the typical early old/new effect, as in Bader et al. (2010). However, the recombined pairs in our paradigm may also elicit some form of fluency or absolute familiarity because they can be very similar to previously studied pairs. For example, if *milk taxi* (a delivery service for dairy products) has been studied, the pair *vegetable taxi* (a possible recombination from 2 different study pairs in our stimulus set) may occur at test, which could describe another kind of *taxi* and may therefore be processed more fluently than completely new pairs. This enhanced processing fluency/baseline familiarity may have wiped out any early intact/recombined ERP differences at parietal recording sites. In line with the idea that both old and recombined pairs are fluently processed, Parks and Yonelinas (2015) reported that in a lexical decision task following the encoding phase of a similar paradigm, old and recombined pairs were more likely to be endorsed as legal compound words than new pairs. Therefore, to distinguish old from recombined pairs in a recognition test, more diagnostic than absolute familiarity may be the relative increment in familiarity due to the encounter in the preceding encoding phase (i.e., relative familiarity), being reflected in the frontal distribution of our ERP effect. 

 As we did not include any new pairs, we cannot directly test this explanation for the different distributions within the same dataset. Notably, Tibon et al. (2014) found that in their unitization condition the old/recombined difference for semantically related picture pairs was frontally distributed as in the present study. In this study, too, absolute familiarity may not have been diagnostic for the old versus recombined decision because old and recombined picture pairs were semantically related (e.g., *desk* and *desk lamp*) and the pairing therefore presumably exhibited high pre-experimental absolute familiarity. It appears therefore that distributions of ERP effects in associative memory tasks encouraging different degrees of unitization can depend on whether the contrast is between old and new, or old and recombined pairs, which differentially bring to light the neural correlates of absolute and relative familiarity signals, respectively. 

Spatial Distribution in the Sentence Condition. In the sentence condition, the early intact/recombined effect exhibited a more posterior maximum and the distribution differed from the intact/recombined effect in the definition condition. A simple explanation for this difference in scalp distributions is that the following late parietal intact/recombined effect, which was only present in the sentence condition, temporally overlapped with the earlier effect, consequently distorting its scalp distribution. It is even possible that the early intact/recombined effect in the sentence condition was entirely driven by a relatively early onset of the following late parietal intact/recombined effect. Nevertheless, based on the data at hand we cannot rule out the alternative explanation that the parietal distribution of the early intact/recombined effect in the sentence condition is a reflection of an absolute familiarity signal.

#### Late Intact/Recombined Effect

 The late parietal intact/recombined effect was observed in the sentence but not in the definition condition. This finding is in line with Bader et al. (2010) and suggests that recollection is recruited when word pairs encoded as inter-item associations are retrieved (e.g., Donaldson & Rugg, 1998), while recollection is not necessary to distinguish old from recombined pairs when the word pairs have been unitized as a single compound word. 

### Contribution of Familiarity and Recollection to Retrieval of Unitized and Non-Unitized Associations

 Our findings add to prior literature that suggests that familiarity can support associative retrieval when the components of an association are unitized. We also replicated the finding that unitization under some circumstances can reduce the late old/new (or intact/recombined) effect reflecting retrieval based on recollection (Bader et al., 2010). Therefore, our data allow for the conclusion that to retrieve word pairs unitized by means of a definition that describes the pair as a novel concept, familiarity is sufficient, while recollection contributes to retrieval of pairs that have been associatively encoded with a weaker level of unitization. This is in accordance with prior reports from neuroimaging studies and studies with patients with hippocampal lesions, showing that successful retrieval in the definition but not the sentence condition can occur without hippocampal involvement (Bader et al., 2014 ; Quamme et al., 2007). 

 Notably, our ERP result patterns (as well as those from Bader et al., 2010) do not perfectly parallel estimates of recollection and familiarity parameters from ROC curves from the same paradigm. For example, Parks and Yonelinas (2015) reported that contributions of both recollection and familiarity were larger in the definition than the sentence condition. While the stimulus set and instructions are not exactly the same in their study and ours (in part due to their participants being speakers of English and ours of German), it is also likely that recollection and familiarity estimates from ERPs and ROC curves do not measure exactly the same underlying phenomena. Nevertheless, the majority of behavioral and ERP studies suggest that unitization affects associative memory mainly by increasing familiarity. 

## Footnotes

1 In Bader et al. (2014) , the words of some pairs were presented in plural form to allow for the reversed pairing to be a legal compound word as well. As no reversed pairs were presented in the present study, we used the singular form whenever possible.

2 The rationale for excluding low confidence recognition judgments (maybe old or maybe new) was that these response options were worded such that they likely contained guesses. However, we repeated the analysis also including the low confidence trials and the results were equivalent.

3 Early old/recombined effect: In the 4 × 2 (Electrode Cluster [left frontal, right frontal, left parietal, right parietal] × Pair Status [old, recombined]) ANOVAs for the definition (*p* > .44 in each case) and the sentence (*p* > .43 in each case) conditions, there were no significant main effects or interactions including the factor pair status. *T*-tests at each electrode cluster also revealed no significant differences between old and recombined pairs in either condition (*p* > .31 in each case). Late old/recombined effect: The ANOVA for the definition condition as well as *t*-tests at each electrode cluster alone revealed neither effects nor interactions including the factor pair status (*p* > .28 in each case). However, in the sentence condition the ANOVA revealed a significant main effect for pair status, *F*(1, 18) = 6.38, *p* = .02, η_p_^2^ = .26.

## Author Note

This research was supported by the International Research Training Group “Adaptive Minds” (IRTG 1457) funded by the German Research Foundation. All procedures were in line with the Declaration of Helsinki and were approved by the local ethics committee. All participants provided informed consent.
